# Differential Support of *Aspergillus fumigatus* Morphogenesis by Yeast and Human Actins

**DOI:** 10.1371/journal.pone.0142535

**Published:** 2015-11-10

**Authors:** Lawrence L. LeClaire, Jarrod R. Fortwendel

**Affiliations:** 1 Department of Biochemistry and Molecular Biology, College of Medicine, University of South Alabama, Mobile, Alabama, United States of America; 2 Department of Microbiology and Immunology, College of Medicine, University of South Alabama, Mobile, Alabama, United States of America; Geisel School of Medicine at Dartmouth, UNITED STATES

## Abstract

The actin cytoskeleton is highly conserved among eukaryotes and is essential for cellular processes regulating growth and differentiation. In fungi, filamentous actin (F-actin) orchestrates hyphal tip structure and extension via organization of exocytic and endocytic processes at the hyphal tip. Although highly conserved, there are key differences among actins of fungal species as well as between mammalian and fungal actins. For example, the F-actin stabilizing molecules, phalloidin and jasplakinolide, bind to actin structures in yeast and human cells, whereas phalloidin does not bind actin structures of Aspergillus. These discrepancies suggest structural differences between Aspergillus actin filaments and those of human and yeast cells. Additionally, fungal actin kinetics are much faster than those of humans, displaying 5-fold faster nucleation and 40-fold faster nucleotide exchange rates. Limited published studies suggest that these faster actin kinetics are required for normal growth and morphogenesis of yeast cells. In the current work, we show that replacement of Aspergillus actin with yeast actin generates a morphologically normal strain, suggesting that Aspergillus actin kinetics are similar to those of yeast. In contrast to wild type A. fumigatus, F-actin in this strain binds phalloidin, and pharmacological stabilization of these actin structures with jasplakinolide inhibits germination and alters morphogenesis in a dose-dependent manner. We also show that human β-actin cannot support Aspergillus viability, even though the amino acid sequences of human and Aspergillus actins are 89.3% identical. Our findings show that minor differences in actin protein sequence account for loss of phalloidin and jasplakinolide sensitivity in Aspergillus species.

## Introduction

Filamentous fungi require striking morphologic plasticity during normal growth and development. Fungal morphology is underpinned by a highly dynamic actin cytoskeleton that is capable of forming a variety of specialized structures including patches, rings, and cables [1, 2]. Formation of these structures allows filamentous fungi to undergo complex developmental growth patterns by: 1) supporting delivery of new cell wall and plasma membrane material to the growing hyphal tip (cables); 2) spatially segregating endocytosis and exocytosis at the hyphal tip, thereby concentrating polarized growth machinery to the apex (patches and cables); and 3) supporting septation to limit the extent of mechanical damage to hyphae (rings). For fungi, these structures were each originally identified in the model yeast, *Saccharomyces cerevisiae*, which has since functioned as an invaluable model for the study of actin dynamics and function for all eukaryotes [3–5]. In many yeast organisms, including *S*. *cerevisiae*, *Schizosaccharomyces pombe*, *Candida albicans*, and *Cryptococcus neoformans*, and in the filamentous fungus, *Ashbya gossypii*, these higher order actin structures can be detected using rhodamine-conjugated phalloidin [6–8]. Phalloidin is a natural compound that binds filamentous actin (F-actin) at the interface of actin subunits, prevents subunit dissociation, and stabilizes the filament [9, 10]. However, multiple filamentous fungi, such as *Aspergillus nidulans* and *Neurospora crassa*, contain an actin cytoskeleton that is insensitive to staining with rhodamine-phalloidin [2, 11]. The mechanistic reason for this loss of phalloidin binding is unknown. Amino acid residues required for phalloidin contact are conserved in *Aspergillus*, as well as in other phalloidin-negative fungi [12]. It has been postulated that amino acid residues surrounding the critical phalloidin contact regions may be involved, as the phalloidin-negative species are not perfectly conserved in these regions. Little attention has been given to the underlying causes of phalloidin insensitivity in filamentous fungi, as immunostaining protocols using fluorophore-coupled, anti-actin antibodies can be employed to visualize the actin cytoskeleton. This method has been useful for detecting both patches and ring structures in *Aspergillus* [13]. Actin cables are not readily identified in these filamentous fungi by immunostaining, but have since been identified using live cell techniques employing the F-actin probe, LifeAct [14–16]. This finding indicates that actin cables in phalloidin-negative filamentous fungi are labile and do not survive the fixation process required for immunostaining.

In accordance with its central role in cellular biology, disruption of the fungal actin cytoskeleton is known to halt polarized growth and alter hyphal morphogenesis. For example, *S*. *cerevisiae* grown in the presence of the monomeric actin binding compound, latrunculin A, has no detectable actin structures and forms large, spherical cells with altered cell walls [17]. The yeast-to-hypha transition in *C*. *albicans* is completely blocked by treatment with either latrunculin A or with another actin polymerization inhibitor, cytochalasin A [18–20]. Further, treatment of *A*. *nidulans* hyphae with cytochalasin A or latrunculin B causes loss of both ring and patch structures leading to decreased growth and swollen hyphal tips [13, 21]. This finding is mirrored in *N*. *crassa* where LifeAct-labeled actin cables rapidly dissipate from the hyphal tip upon latrunculin A treatment leading to diminished germ tube elongation [14]. Mechanistically, latrunculin compounds bind to actin monomers to inhibit their incorporation into actin filaments, whereas cytochalasins bind directly to ends of actin filaments and block the further addition of actin monomers [22, 23]. Both of these compounds have the overall effect of blocking actin polymerization leading to loss or delocalization of F-actin structures required to promote polarized morphogenesis. In yeast, not only the inhibition of polymerization but also the promotion of F-actin polymerization has been studied for its impact on hyphal growth and morphology. This has been accomplished using jasplakinolide, a cell-permeable F-actin stabilizing compound [24]. Jasplakinolide is a cyclo-depsipeptide that was originally isolated from the marine sponge, *Jaspis johnstoni* [25]. Like phalloidin, jasplakinolide has been shown to be effective at stabilizing F-actin in a wide variety of organisms, including mammalian and fungal cells [26, 27]. In both *S*. *cerevisiae* and *C*. *albicans*, decreasing actin dynamics via jasplakinolide treatment blocks the generation of cell polarity [3, 20]. In *S*. *cerevisiae*, this block in polarity establishment is coupled with decreased levels of fluid-phase endocytosis, suggesting multiple actin-based mechanisms are inhibited by decreased actin dynamics [3]. No studies regarding jasplakinolide sensitivity of filamentous fungi have been reported. Because jasplakinolide and phalloidin share a common F-actin biding pocket [24], and actin structures of some filamentous fungi are not phalloidin-sensitive, it is likely that these organisms are also insensitive to jasplakinolide. However, any potential jasplakinolide insensitivity in these fungi might also be explained by lack of cytoplasmic availability. For example, in *S*. *cerevisiae*, deletion of multidrug resistance ABC transporters is required to generate reasonable jasplakinolide sensitivity [3].

Using an *A*. *fumigatus* mutant that expresses *S*. *cerevisiae* Act1p as the sole source of cellular actin, we show that pharmacological stabilization of F-actin structures with jasplakinolide inhibits establishment of polarity, alters hyphal morphology, and induces cell wall abnormalities. These phenotypic outcomes are similar to earlier published studies in *A*. *nidulans*, described above, that have employed cytochalasin and latrunculin. However, the effect of F-actin stabilization on overall actin cytoskeleton structure is remarkably different. Jasplakinolide treatment of the *S*. *cerevisiae* Act1p expressing strain caused aggregation of actin into large clumps distributed along hyphae. In contrast to *S*. *cerevisiae*, deletion of multidrug resistance transporters was not required to induce jasplakinolide sensitivity. We also show that human β-actin, expressed as the sole source of cellular actin, cannot support *A*. *fumigatus* viability. Taken together, our results suggest that minor differences in actin protein sequence dictate loss of jasplakinolide sensitivity in *A*. *fumigatus* and that the rapid turnover of F-actin, characteristic of the dynamic fungal actin cytoskeleton, is required for normal development of invasive hyphae.

## Materials and Methods

### Strains, culture conditions and growth rate analyses

The *Aspergillus fumigatus* wild type H237 and mutant strains were maintained on *Aspergillus* Glucose Minimal Medium (GMM) [28]. For analysis of colony morphology and for quantification of radial growth rates, 1 X 10^4^ conidia were inoculated in triplicate onto GMM or YPD (10% w./v. yeast extract, 20% w./v. peptone, 20% w./v. dextrose) solid agar and incubated at 30°C or 37°C, for the indicated times. Colony diameter was measured every 24 hours and results reported as the average diameter in millimeters ± standard deviation. For the *HsactB* heterokaryon, radial growth analysis was performed in a similar manner, with the exception that GMM solid agar plates were inoculated with agar plugs containing hyphae from the colony periphery of previous GMM culture plates. For germination studies, strains were inoculated into triplicate GMM broth cultures, with or without jasplakinolide (Tocris Cat. No. 2792), at a density of 5 X 10^5^ conidia / ml and incubated at 37°C. Germ tube production was scored microscopically at the indicated times. DMSO was utilized as a control. Data are presented as average percent (%) germination ± standard deviation for each strain.

### Generation of *Scact1* and *HsactB* mutants

To generate *A*. *fumigatus* strains expressing the *S*. *cerevisiae* actin gene (*Scact1*) or the human β-actin gene (*HsactB*) as the sole source of cellular actin, the coding sequences of both yeast and human actin genes were PCR amplified and cloned into an expression vector for homologous integration into the *A*. *fumigatus* H237 wild type strain. In brief, the yeast actin gene (GeneID:850504) was amplified from *S*. *cerevisiae* genomic DNA using primers ACT1 Forward (TTTTGCGGCCGCATGGATTCTGAGGTTGCTGCTTTGGTTATTG) and ACT1 Reverse (TTTTGCGGCCGCTTAGAAACACTTGTGGTGAACG). The human β-actin coding sequence (GeneID:60) was PCR amplified from plasmid pCAG-mGFP-Actin, a gift from Ryohei Yasuda (Addgene plasmid #21948) [29], using primers ActB Forward (TTTTGCGGCCGCATGGATGATGATATCGCC) and ActB Reverse (TTTTGCGGCCGCCTAGAAGCATTTGCGGTG). *NotI* restriction sites, underlined in the primer sequences, were utilized for cloning into vector pAEV. Expression vector pAEV was constructed for homologous genomic integration by cloning the promoter region of the *A*. *fumigatus actA* gene (Afu6g04740), to include 1.5 kb upstream of the *actA* start site, into vector pUCGH [30] as a *BamHI* / *NotI* fragment. To complete vector pAEV, 1.5 kb of genomic sequence downstream of the *actA* stop codon was cloned into the previously constructed vector as a *HindIII* fragment. Cloning of actin coding sequences into the *NotI* restriction site of this plasmid produces a vector where actin expression is under control of the endogenous *A*. *fumigatus actA* promoter and allows for selection of integration using hygromycin resistance. The human β-actin gene or yeast *ACT1* were finally cloned into pAEV, screened for proper orientation and then sequenced (ACGT, Inc.) to ensure no errors were introduced during PCR amplification. The full deletion cassette was then PCR amplified from each resulting construct and introduced into the *A*. *fumigatus* H237 wild type, using standard protoplasting methods. Transformants were selected on hygromycin containing media and homologous recombination confirmed via PCR and southern blot analysis.

### Western blot analysis

Western blot analysis for expression of Act1p in the *Scact1* mutant was performed as previously described [30]. Briefly, the H237 and *Scact1* strains were inoculated into GMM broth and incubated for 24 hours at 37°C with shaking at 250 rpm. Hyphae were harvested by vacuum filtration, frozen under liquid nitrogen and crushed to a fine powder with a mortar and pestle. Each sample was then suspended in protein extraction buffer (50mM Tris-HCl [pH 7.5], 150 mM NaCl, 50 mM KCl, 0.01% Triton X-100, 1mM phenylmethylsulfonyl fluoride [PMSF], and 1:100 protease inhibitor cocktail) and subjected to centrifugation for 10 minutes at 4000 rpm to clear cell wall debris. The resulting crude lysate (supernatant) was removed and total protein was quantified utilizing a Bradford assay. Fifty micrograms of total protein was resolved on a 12% SDS-PAGE gel and transferred to a PVDF membrane. Immune blotting was performed using an anti-actin primary antibody (MAB1501, Millipore) coupled with an HRP-conjugated secondary and SuperSignal West Pico substrate (Thermo). For a loading control, fifty micrograms of total protein was resolved on a separate SDS-PAGE gel and stained with Coomasie Brilliant Blue (Bio-Rad).

### Phalloidin staining and fluorescence microscopy

Rhodamine-phalloidin staining was performed as described previously for *A*. *gossypii*, with modification [6]. Briefly, *A*. *fumigatus* strains *w*ere cultured in GMM for 14 hours at 37°C with shaking at 250 rpm. One milliliter of the culture was mixed with 100 μl of 37% formaldehyde and incubated at room temperature for 10 minutes. *A*. *fumigatus* mycelia were then centrifuged at 2000 × *g*, resuspended in phosphate-buffered saline (PBS) containing 4% formaldehyde, and incubated for 1 hour. Mycelia were washed twice with PBST (PBS containing 0.03% Triton X-100) and resuspended in 100 μl of PBST. Ten microliters of rhodamine-phalloidin (Life Technologies, Inc) was added, and the mycelia were incubated for 1 hour in the dark at room temperature. Mycelia were washed 5 times in PBST and mounted for fluorescence microscopy.

### Fluorescent detection of the cell wall, nuclei, and actin structures

Staining of the cell wall was performed as previously described, with slight modification [31]. Freshly harvested conidia from the wild type and *Scact1* strains were inoculated onto cover slips immersed in GMM liquid media. After 16 hours incubation at 37°C, cover slips were removed and inverted onto a 0.5 ml drop of GMM containing 50 μg/ml of jasplakinolide and allowed to incubate for 2 hours at 37°C. After jasplakinolide treatment, cover slips were removed and washed twice in 50 mM PIPES (pH 6.7) and fixed in 5 ml of fixative solution (8% formaldehyde in 50 mM PIPES [pH 6.7], 25 mM EGTA [pH 7.0], 5 mM MgSO_4_, and 5% DMSO) for 40 min. After fixation, the cover slips were washed twice for 10 minutes in 50 mM PIPES (pH 6.7) and inverted onto 0.5 ml of freshly made Fluorescent Brightener 28 [0.4 μg/ml; Sigma] in 50 mM PIPES [pH 6.7]) for 5 minutes at room temperature. Finally, the cover slips were washed twice for 10 minutes in 50 mM PIPES (pH 6.7) and mounted for microscopy.

Immunostaining of actin structures was performed as above, with the following modifications. After fixation, cover slips were washed in 50 mM PIPES (pH 6.7) and then inverted onto a 0.5 ml drop of cell wall digestion solution (50 μg/ml of Vinotaste Pro [Novozymes], 1.2 M MgSO_4_) for 1 hour at room temperature. After cell wall digestion, cover slips were washed twice in 50 mM PIPES (pH 6.7) and inverted onto a 0.5 ml drop of 50 mM PIPES (pH 6.7) containing an anti-actin primary antibody. Cover slips were incubated at room temperature for 1 hour, washed as before, and then inverted onto a 0.5 ml drop of 50 mM PIPES (pH 6.7) containing a TRITC-coupled, secondary antibody and incubated overnight at room temperature. After incubation, cover slips were washed and mounted for microscopy. To stain nuclei, after the final wash step, cover slips were incubated in 50 mM PIPES (pH 6.7) containing 10 μg/ml of Hoechst 33342 (Life Technologies) for 15 minutes before mounting for microscopy.

All fluorescence images were captured on a Nikon U microscope equipped with DAPI (4′,6′-diamidino-2-phenylindole) and mCherry filter cubes and an Intensilight Epi-Fluorescence Illuminator (Nikon). Digital images were analyzed using Nikon Elements Advanced Research software.

## Results

### The *Saccharomyces cerevisiae* actin gene, *ACT1*, is fully functional for *A*. *fumigatus* growth

**Fig 1:**
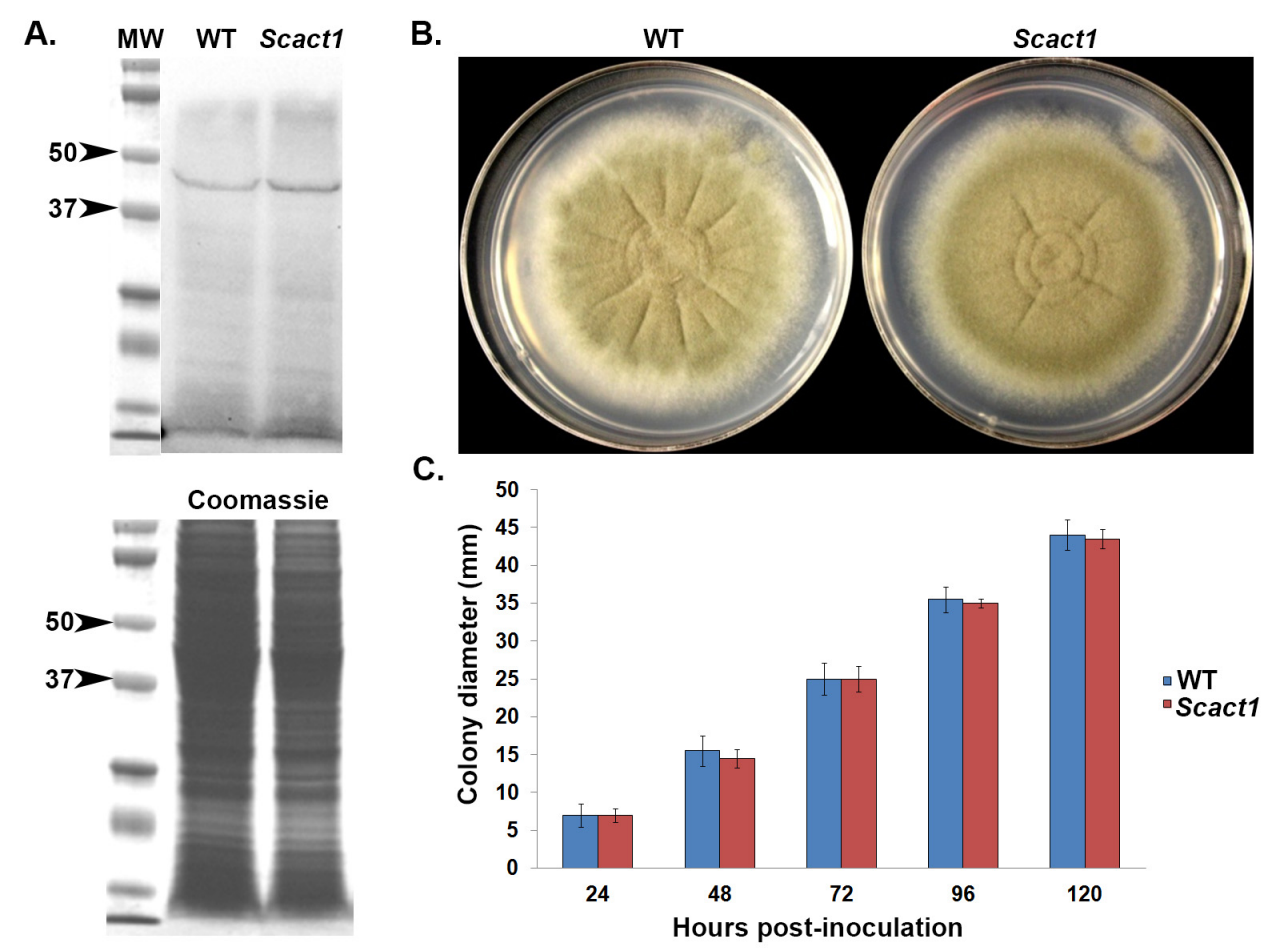
Yeast actin supports growth and viability of *A*. *fumigatus*. (A) Western blot analysis of actin protein expression levels in the wild type (WT) and *S*. *cerevisiae ACT1* expressing strains (*Scact1*). Total protein lysates from 24 hr submerged cultures were separated by SDS-PAGE and detected with an anti-actin antibody (~42 kDa band). Coomassie stained total protein is shown as a loading control. (B) Representative cultures comparing growth and colony morphology of the WT and *Scact1* strains. Conidia were point inoculated onto the center of each GMM agar plate and incubated for up to 5 days at 37°C. (C) Quantification of radial outgrowth (colony diameter) over 120 hrs post inoculation growth. Data represents the average of three experiments ± standard deviation.

To confirm functional similarity between yeast and *Aspergillus* actin, we first generated an *A*. *fumigatus* strain expressing *S*. *cerevisiae* Act1p as the sole source of cellular actin. This strain was generated by replacing the endogenous *A*. *fumigatus actA* coding region with the coding sequence of yeast *ACT1*. This new strain, termed *Scact1*, expresses yeast *ACT1* under the control of the endogenous *A*. *fumigatus actA* promoter. Because any phenotype produced in the *Scact1* strain might be an artifact caused by alterations in actin expression levels, we first performed western blot analysis on the wild type and *Scact1* strains to analyze total actin protein levels. Using an anti-actin primary antibody, western blot analysis revealed that the yeast Act1p protein was present in lysates of the *Scact1* strain at levels similar to that of ActA in wild type *A*. *fumigatus* (Fig 1A). These results suggest that phenotypes produced by the *Scact1* strain are not an artifact of altered cellular actin levels. To determine if the yeast actin gene can support *A*. *fumigatus* filamentous growth, we examined gross colony morphology and diameter of the wild type and *Scact1* mutant. Radial growth analysis revealed that the *Scact1* strain produced an overall colonial morphology with radial growth rates similar to the wild type *A*. *fumigatus* parental strain (Fig 1B and 1C). The similarity between wild type and *Scact1* was independent of external factors affecting growth rate, including temperature and media composition (S1 Fig). In addition, the total number of conidia produced by each strain was similar when cultured at 37°C on minimal media (data not shown).

**Fig 2:**
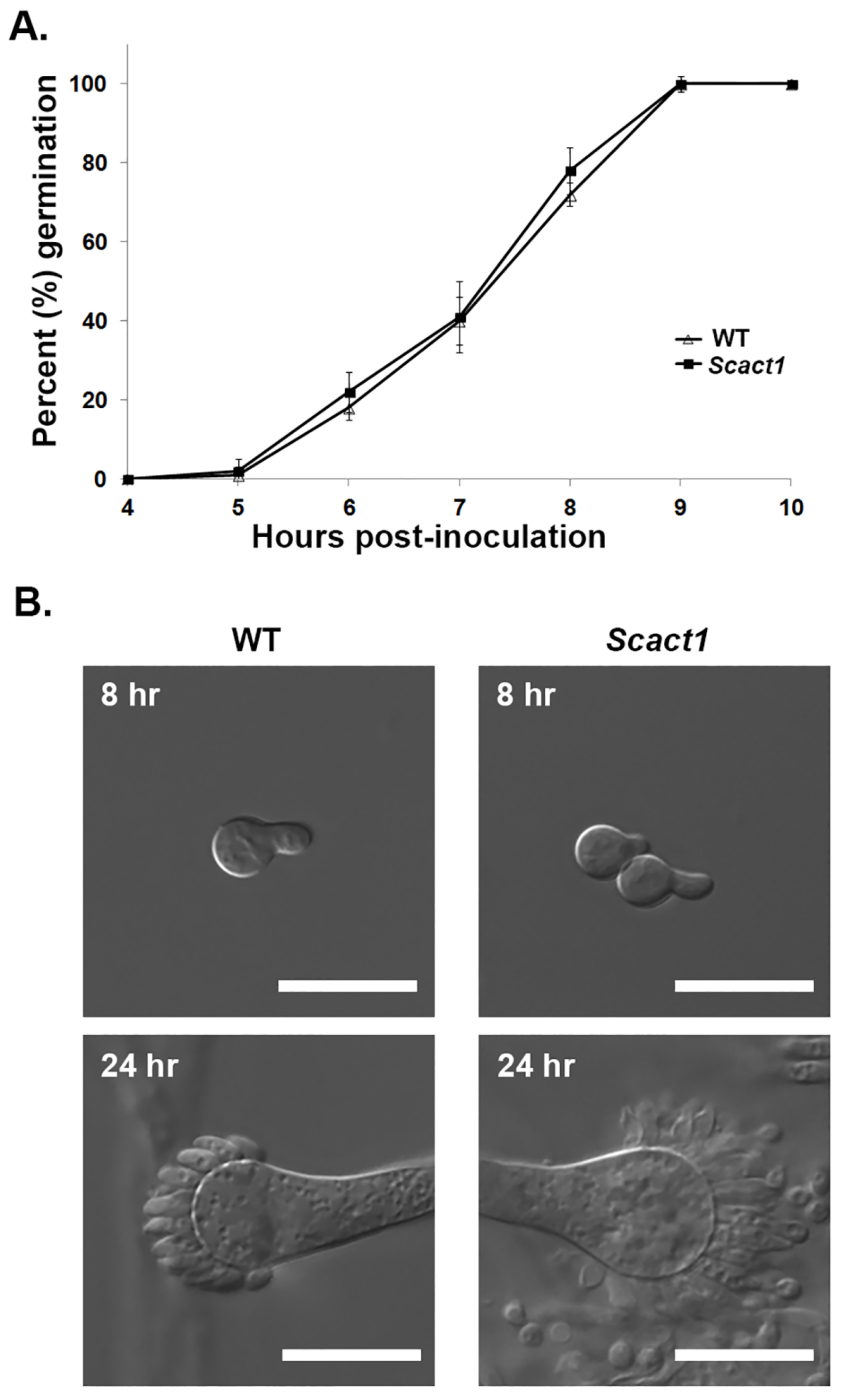
Yeast actin supports normal polarity establishment and asexual development of *A*. *fumigatus*. (A) Germination rates of the WT and *Scact1* strains are identical. Conidia from each strain were inoculated over cover slips submerged in GMM. Beginning at 4 hrs post-inoculation, cover slips were removed and the number of germ tube producing conidia were scored microscopically. Data represents the average of three experiments ± standard deviation. (B) The WT and *Scact1* strains display similar morphologies throughout development. Representative 8 hr germlings (top panels) and 24 hr conidiophores (bottom panels) are shown for the WT and *Scact1* strains. Scale bar = 10 μm.

Morphological characteristics of growth and development of the *Scact1* strain were also examined to ensure full complementation of *actA* substitution. Germination analysis revealed that the wild type and *Scact1* strains produced polarized growth axes at identical rates in minimal medium at 37°C (Fig 2A). Further, the morphology of germ tube formation in the *Scact1* strain was similar to wild type, forming germ tubes of similar length after 8 hours of culture at 37°C (Fig 2A). To see if the later stages of asexual development are temporally or morphologically altered in the *Scact1* mutant, we next compared conidiophore structures in the wild type and *Scact1* strains. After 24 hours of culture at 37°C on minimal media, no differences in *Scact1* conidiophores were noted in comparison to the wild type strain (Fig 2B). This finding, coupled with the wild type levels of conidia production in the *Scact1* strain, indicate that asexual development is not affected by expression of yeast actin as the sole source of cellular actin protein in *A*. *fumigatus*. Taken together, our phenotypic data show that yeast actin is fully competent to support hyphal growth and morphogenesis in a filamentous fungus.

### F-actin structures of the *A*. *fumigatus Scact1* mutant binds phalloidin

**Fig 3:**
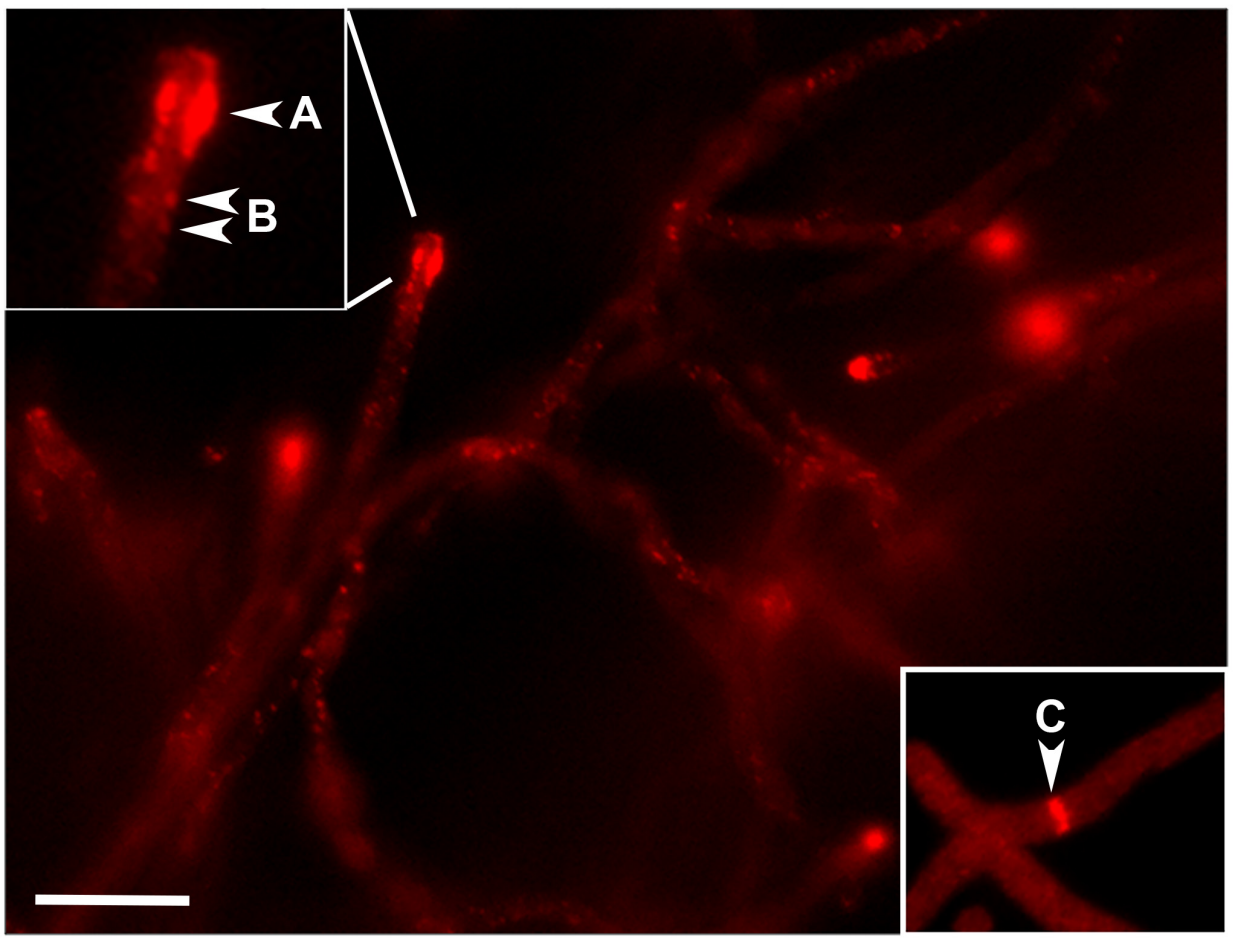
Actin structures of *Scact1* stain with rhodamine-phalloidin. Staining of *Scact1* hyphae with rhodamine-phalloidin detected actin patches and rings. Actin patches were arranged as a sub-apical actin collar (A), as previously identified in filamentous fungi, and also found positioned along the hyphal cortex (B). Actin rings (C) were detected at sites of newly forming septa. Staining of the WT strain with rhodamine-phalloidin produced no detectable fluorescent signal (data not shown). Scale bar = 50 μm.

As previously stated, actin structures produced by multiple filamentous ascomycetes are not sensitive to staining with rhodamine-phalloidin [2, 32]. In contrast, *S*. *cerevisiae* F-actin is readily detected by a fluorophore-coupled phalloidin probe [33]. To see if expression of yeast actin is sufficient to permit phalloidin binding of F-actin structures in *A*. *fumigatus*, we next performed fluorescent labeling with rhodamine-phalloidin. F-actin filaments of the *A*. *fumigatus* wild type strain were not detectable with rhodamine-phalloidin in our studies, as absolutely no fluorescent signal was detected after fixation and staining (data not shown). These findings are in agreement with previous work showing that *Aspergillus* actin structures are phalloidin negative [12, 34]. In contrast, *Scact1* F-actin filaments stained intensely with rhodamine-phalloidin employing protocols commonly used for yeast and the filamentous fungus, *Ashbya gossypii* [6]. Rhodamine-phalloidin staining detected hyphal-tip polarized actin, forming a sub-apical endocytic ring and cortical actin patches in sub-apical compartments (Fig 3). Cytokinetic actin rings were also identifiable at sites of newly forming septa (Fig 3 inset). Therefore, yeast actin expressed in *A*. *fumigatus* is capable of forming the normal, highly dynamic F-actin structures typical of filamentous fungi [2]. Further, our data show that expression of actin from a phalloidin-positive species is sufficient to confer phalloidin binding in a previously phalloidin-negative fungal species.

### Stabilization of F-actin inhibits *A*. *fumigatus* growth

Jasplakinolide is a cell-permeable, actin stabilizing compound that competes with phalloidin for F-actin binding, suggesting a similar mechanism for F-actin stabilization [24]. Jasplakinolide treatment disrupts cellular morphology and causes alterations of actin dynamics in the yeast organisms, *S*. *cerevisiae* and *C*. *albicans* [3, 19, 35]. Although the lack of phalloidin staining in wild type *A*. *fumigatus* may imply a lack of sensitivity to jasplakinolide, no published studies have reported the sensitivity of *Aspergillus* species to jasplakinolide treatment. We, therefore, predicted that *A*. *fumigatus* growth would not be inhibited by jasplakinolide, whereas pharmacological alteration of actin dynamics and growth inhibition would be achievable in the *Scact1* strain.

**Fig 4:**
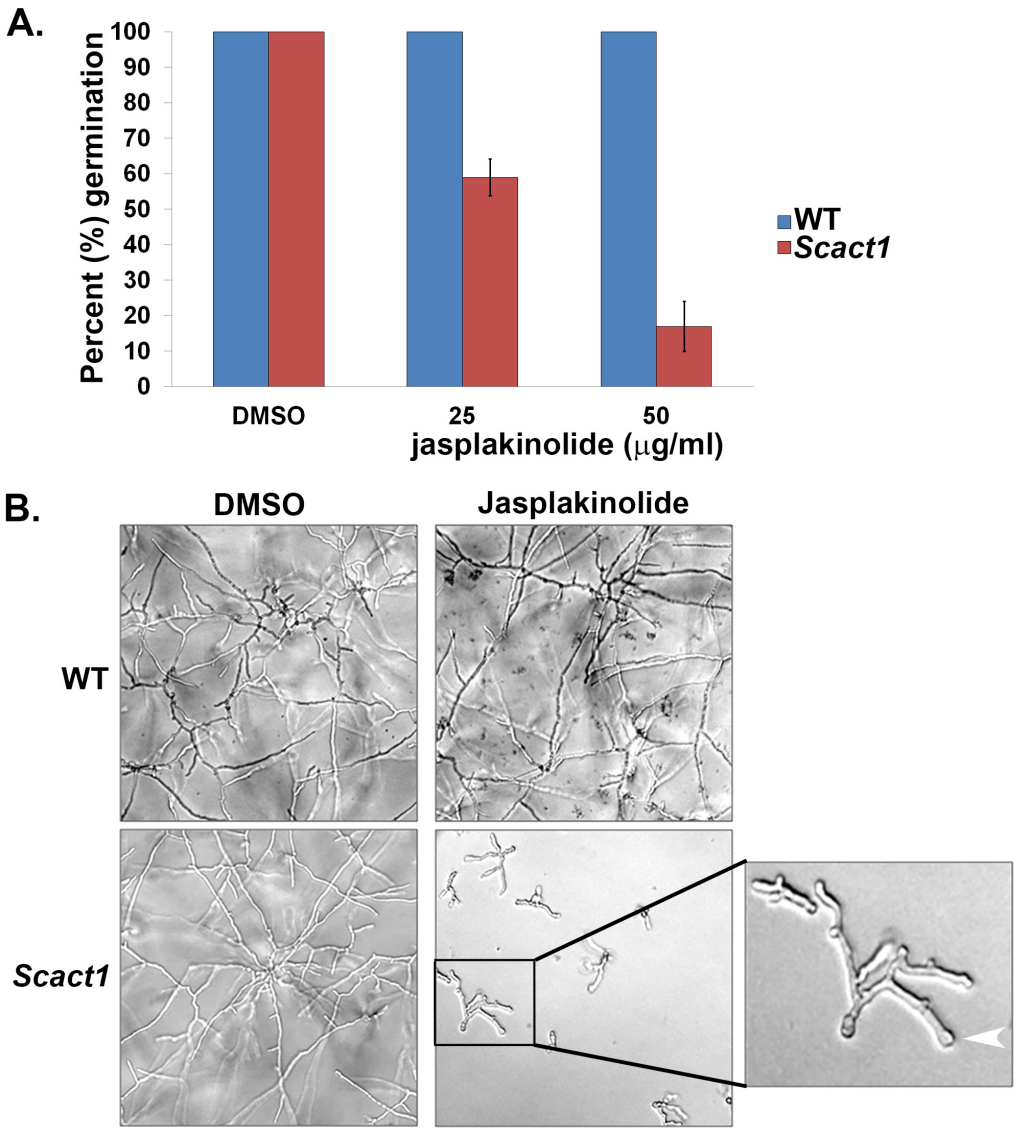
The *Scact1* strain is sensitive to the F-actin stabilizing agent, jasplakinolide. (A) Jasplakinolide treatment inhibits *Scact1* germination in a dose dependent manner. Conidia from the WT and *Scact1* strains were cultured in the presence of increasing doses of jasplakinolide and scored for germ tube formation after 12 hrs incubation at 37°C. Data are presented as the average of three experiments ± standard deviation. (B) Jasplakinolide inhibits growth and morphogenesis of the *Scact1* but not WT strain. Conidia were inoculated into a multi-well plate containing liquid GMM and ascending concentrations of jasplakinolide and incubated for 24 hrs at 37°C. Effects on WT and *Scact1* growth at 50 μg/ml jasplakinolide are shown. Jasplakinolide treatment was associated with decreased growth, increased branching and swollen hyphal tips (white arrowhead, enlarged panel to right).

To test this hypothesis, we cultured conidia of the wild type and *Scact1* strains in the presence of ascending doses of the F-actin stabilizing compound. As predicted, germination and hyphal growth of the *A*. *fumigatus* wild type strain was not inhibited, even at the highest concentration of jasplakinolide (Fig 4A and 4B). In contrast, germ tube formation and hyphal growth of the *Scact1* strain were inhibited by jasplakinolide treatment in a concentration dependent manner. After 12 hours of culture at 37°C, germination of the *Scact1* strain was inhibited by 40% at 25 μg/ml of jasplakinolide (Fig 4A). Strikingly, *Scact1* germination was inhibited by almost 90%, compared to the non-treated control, when cultured in the presence of 50 μg/ml jasplakinolide (Fig 4A). After 24 hours of culture at 37°C, morphological aberrations in the *Scact1* mutant treated with jasplakinolide included the formation of stunted hyphae with increased branching and the production of swollen hyphal tips (Fig 4B). These findings suggested treatment with jasplakinolide produced a loss of polarity and altered cell wall integrity.

**Fig 5:**
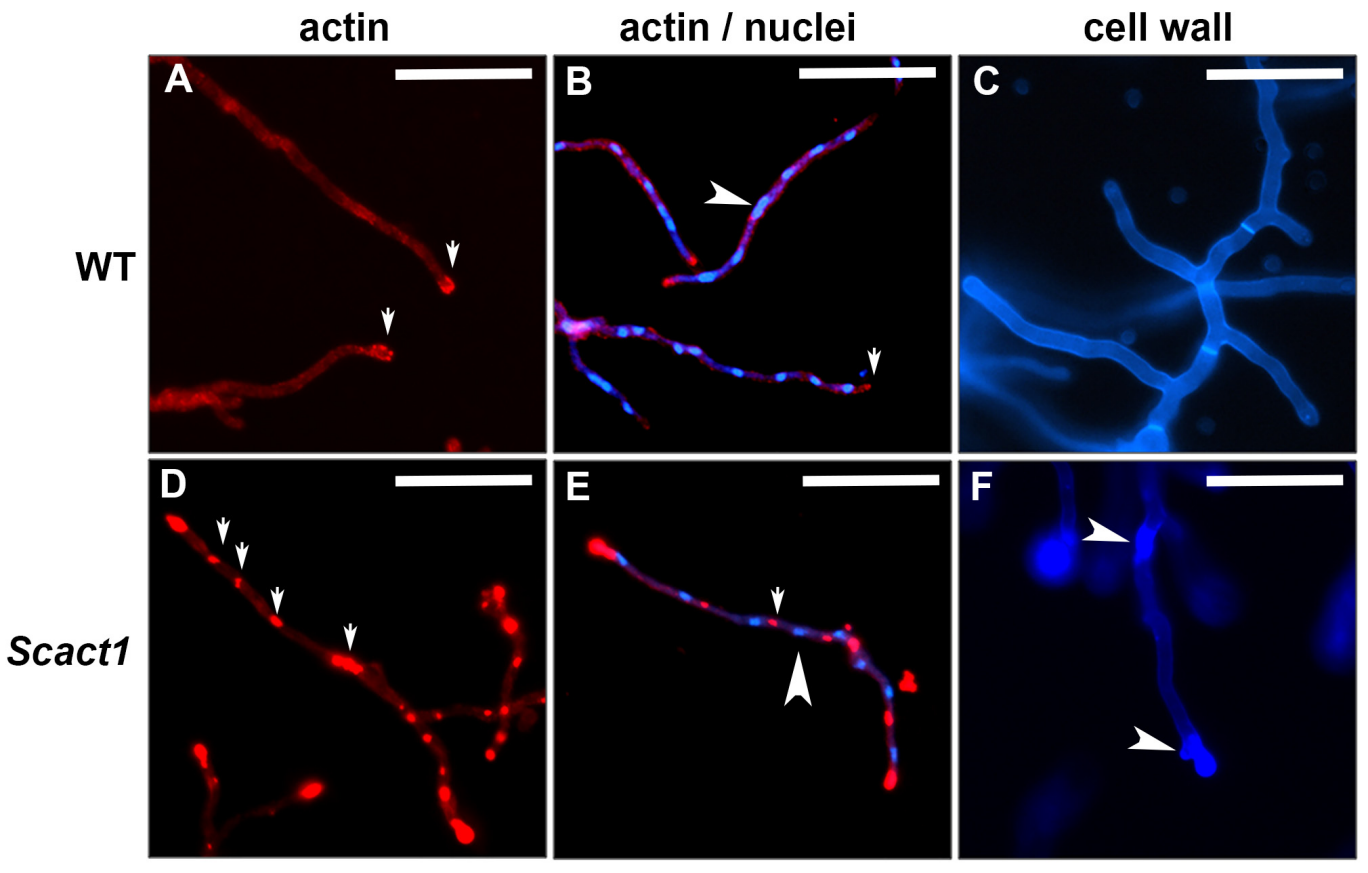
F-actin stabilization alters actin dynamics and cell wall construction in *A*. *fumigatus*. Conidia of the WT and *Scact1* strains were inoculated onto cover slips submerged in GMM and incubated for 24 hr at 37°C. Adherent hyphae were subsequently treated with jasplakinolide (50 μg/ml) for 2 hours at 37°C. Cultures were fixed and immunostained with an anti-actin antibody (red) either alone (A and D) or in combination with Hoechst (blue) to detect nuclear position (B and E). White, block arrows indicate areas of actin structure accumulation. Note normal polarization of the cytoskeleton to the hyphal tip in the WT strain in the presence of jasplakinolide (A and B) and the disorganization of aggregated actin into clumps in the jasplakinolide-treated *Scact1* strain (D and E). Small white arrowheads denote nuclei (B and E). To detect changes in cell wall deposition, calcofluor white staining was performed on unfixed samples treated with 50 μg/ml jasplakinolide (C and F). White arrowheads denote areas of aberrant cell wall deposition in the jasplakinolide-treated *Scact1* strain (C and F). Scale bar = 50 μm.

To confirm that treatment is associated with changes in F-actin dynamics, the wild type and *Scact1* strains were germinated on cover slips and subsequently exposed to jasplakinolide for two hours. After treatment, each strain was immunolabeled with an anti-actin antibody to reveal actin structures. The jasplakinolide-treated wild type strain displayed typical actin dynamics, including tip-concentrated actin coupled with sub-apical cortical actin patches (Fig 5A). In contrast, upon exposure to jasplakinolide, the *Scact1* mutant displayed large, intensely stained aggregates of actin located to the hyphal tip and regularly spaced along the length of each hypha (Fig 5D). The regular spacing of the actin aggregates somewhat resembled the distribution of nuclei along *Aspergillus* spp. hyphae [36, 37]. Because, perinuclear F-actin structures have recently been identified in cultures of *Cryptococcus neoformans* treated with microtubule inhibitors [38], we co-stained jasplakinolide-treated strains for actin and nuclei to examine possible co-localization of these structures in the *Scact1* mutant. Hoechst staining revealed normally spaced nuclei in the wild type strain upon F-actin stabilization (Fig 5B). Spacing of nuclei in the *Scact1* mutant treated with jasplakinolide also appeared normal and nuclei never co-localized with actin aggregates. Instead, nuclei were interspersed with the aggregated actin structures (Fig 5E). Calcofluor white staining further revealed altered cell wall structure in the *Scact1* mutant after F-actin stabilization. In contrast to the homogenous cell wall staining revealed in the wild type strain (Fig 5C), the *Scact1* mutant displayed patches of intense cell wall staining at hyphal tips and in sub-apical compartments after jasplakinolide treatment (Fig 5F).

### Human β-actin does not support *A*. *fumigatus* viability

**Fig 6:**
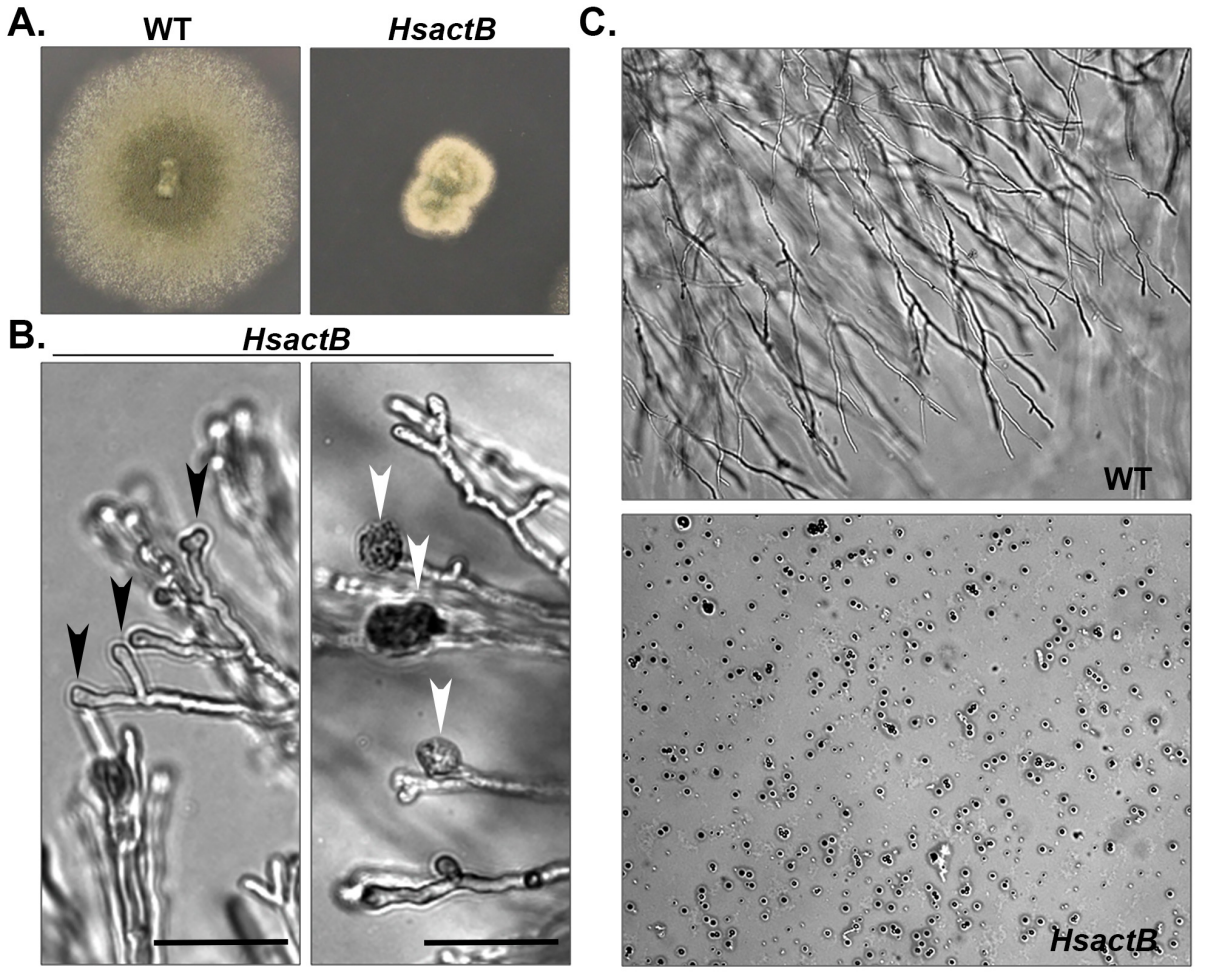
Human β-actin does not support *A*. *fumigatus* viability. (A) Hyphal plugs from the wild type and *HsactB* heterokaryon strains grown for 48 hrs at 37°C on GMM. Cores of agar containing hyphae taken from the colony periphery of previous cultures where transferred to new GMM agar plates for culture. (B) Hyphae of the *HsactB* heterokaryon growing on GMM agar plates after 48 hrs at 37°C. Blunted hyphal tips (black arrowheads) that regularly lysed (white arrowheads) were noted. Scale bar = 50 μm (C) Conidia harvested from each cultures represented in Panel A were inoculated onto hygromycin selective agar and cultured for 48 hrs at 37°C. Note the lack of germination of the *HsactB* heterokaryon strain under selection, indicating inability of human actin to support *A*. *fumigatus* viability.

The phenotypes produced by jasplakinolide treatment suggested that growth and morphogenesis of *A*. *fumigatus* is highly sensitive to pharmacological inhibition of F-actin turnover. To explore this at the molecular level, we attempted to generate an *A*. *fumigatus* mutant expressing the human β-actin gene as the sole source of cellular actin. Human actin is known to have slower F-actin turnover when compared to fungal F-actin filaments [39, 40]. Using a strategy identical to the *Scact1* mutant described above, a strain expressing only human β-actin could not be obtained, suggesting this was a lethal substitution. Fortunately, a heterokaryon strain, termed *HsactB*, expressing both the endogenous *A*. *fumigatus actA* gene and the newly integrated human β-actin gene, was recovered for further analysis. Comparison of colony morphology and radial growth rates from transplanted agar plugs of the wild type and *HsactB* heterokaryon revealed the *HsactB* heterokaryon to be a slow growing strain with reduced conidiation (Fig 6A). In addition, growth rates of the wild type and *HsactB* strain increased in response to temperature shift from 30°C to 37°C and when cultured on rich media versus defined media (S1 Fig). These results suggested F-actin of the *HsactB* heterokaryon is able to support changes in growth rate induced by external factors. Microscopic examination of *HsactB* cultures revealed the development of stunted hyphae with swollen hyphal tips exhibiting frequent lysis (Fig 6B). Similar aberrant hyphal morphology was not seen in the wild type strain under identical growth conditions. Conidia produced by the heterokaryon were isolated and subsequently cultured onto hygromycin containing media, resulting in complete lack of growth (Fig 6C). This finding confirmed the inability of human β-actin to support *Aspergillus* growth, as uninucleate conidia produced by the heterokaryon strain either harbored wild type nuclei (and therefore were inhibited under hygromycin selection) or harbored mutant nuclei expressing the human β-actin gene (and were therefore inviable). Taken together, these data suggest that human β-actin is unable to support hyphal morphogenesis and cell wall construction during *Aspergillus* growth.

## Discussion

The dynamic localization of actin cytoskeleton proteins to specific cellular compartments during filamentous fungal development is well characterized [2]. To date, the importance of a functional actin cytoskeleton to *Aspergillus* morphogenesis has largely been ascertained through multiple studies using actin polymerization inhibitor compounds, like cytochalasin and latrunculin. However, the importance of dynamic filament turnover and rapid actin assembly kinetics to filamentous fungal growth and morphogenesis has remained largely unexplored. Previous studies show that actin isoforms from fungi, protozoa, and mammals possess different kinetic properties such as rates of assembly, disassembly, and adenosine triphosphate (ATP) exchange. For example, yeast actin assembly kinetics have been studied extensively and are known to be faster than human actin assembly [39–41].

The assembly kinetics of filamentous fungal actin and turnover are completely unknown. However, actin from *S*. *cerevisiae* is 92% identical to that of *A*. *fumigatus* and shown to have the faster filament assembly and turnover kinetics associated with yeast actins [40–42]. In addition, yeast F-actin filaments are stainable with rhodamine-phalloidin and sensitive to the stabilizing effect of jasplakinolide [7]. To generate an *A*. *fumigatus* strain in which we could study the effects of stabilizing F-actin structures, we replaced the native *actA* gene with the actin gene from the budding yeast, *S*. *cerevisiae*. The *Scact1* strain grew identically to the wild type under normal culture conditions, suggesting that *A*. *fumigatus* and *S*. *cerevisiae* F-actin assembly kinetics are similar. Using protocols designed for yeast, actin filaments of the *Scact1* strain labeled strongly with rhodamine-phalloidin whereas the actin filaments of the wild type remained unstained. This finding proved that access to the phalloidin binding site is preserved in our *Scact1* mutant. Therefore, sensitivity of *A*. *fumigatus* to jasplakinolide could be explored. Treatment with jasplakinolide, which competes for the same filament binding site as phalloidin [24], prevented the establishment of polarity and altered morphogenesis of the *Scact1* strain in a dose dependent manner while not affecting the wild type. This phenotype is mirrored in jasplakinolide treated yeast, where F-actin stabilization blocks budding and polarity establishment in both *S*. *cerevisiae* and *C*. *albicans* [3, 20]. Because nascent cell wall material is incorporated at the hyphal apex during extension (reviewed in [21, 43]), it is logical to assume the inhibition of polarized growth via F-actin stabilization should lead to an altered deposition of cell wall materials. In support of this, calcofluor white staining revealed aberrant cell wall material present at the tips *Scact1* hyphae treated with jasplakinolide.

Jasplakinolide caused the aggregation of *Scact1* actin proteins at the tip and along the length of hyphae. The underlying cause of the regular spacing of actin aggregates noted in the *Scact1* strain after jasplakinolide treatment is unknown and will be the focus of future study. However, the jasplakinolide-induced re-organization of actin into these aggregates is similar to that reported for jasplakinolide treated *S*. *cerevisiae* cells. In the presence of jasplakinolide, yeast actin forms a single, large aggregate that exists stably in the F-actin form [3]. Actin subdomain organization within these aggregated yeast actin filaments is preserved and actin protein binding sites are still accessible, suggesting jasplakinolide treatment does not overly perturb F-actin structure [3]. Therefore, the effects of jasplakinolide are largely attributable to the decreased turnover of F-actin filaments and not complete disruption of cytoskeletal proteins. As mentioned previously, fungal actin kinetics are much faster than human actin kinetics, displaying 5-fold faster nucleation and 40-fold faster nucleotide exchange rates in yeast [39, 40]. To generate an *A*. *fumigatus* mutant with decreased actin kinetics, we attempted to create a strain in which the *A*. *fumigatus* actin gene was replaced with the human β-actin gene. However, although human β-actin and *A*. *fumigatus* actin are 89% identical, human β-actin could not support *A*. *fumigatus* viability. Only heterokaryon mutants, developing stunted hyphae with apical compartments that regularly lysed, could be obtained. This phenotype denotes a potential cell wall integrity defect that is similar to the jasplakinolide treated *Scact1* strain.

This work corroborates previously published experiments showing that actin from higher eukaryotes does not support viability in the yeast organism, *S*. *cerevisiae*. For example, mammalian muscle actin is unable to support *S*. *cerevisiae* growth [44]. Additionally, although it was originally reported that chicken β-actin is able to support *S*. *cerevisiae* viability under normal growth conditions, closer examination of the data reveals this conclusion to be tenuous [45]. Replacement of yeast *ACT1* with chicken β-actin produces a yeast mutant that is inviable at 37°C, is slow growing at 30°C, and displays altered morphogenesis, characterized by enlarged cell size [45]. Together, these findings have laid the groundwork to prove that rapid dynamics of fungal actin are essential for supporting the normal growth and morphogenetic processes required for fungal viability. Because morphogenesis is intimately tied to virulence in a monomorphic mold like *A*. *fumigatus*, the requirement for rapid actin cytoskeleton dynamics may represent a pressure point in fungal virulence. Our data support the hypothesis that complete disruption of F-actin turnover is unnecessary to inhibit *Aspergillus* viability. Rather, reducing F-actin kinetics to those of human actins may be enough to inhibit fungal viability and invasion.

Due to the high conservation between yeast and *Aspergillus* actin proteins, our data also suggest that very minor, yet significant, differences in actin amino acid sequence account for the observed loss in phalloidin binding and jasplakinolide sensitivity. Amino acids critical for phallotoxin binding have been identified in *S*. *cerevisiae* [46] and these residues are largely conserved in *A*. *fumigatus* [32]. However, as previously noted, amino acid residues surrounding the phalloidin pocket are not absolutely conserved [32]. It is reasonable to hypothesize that these changes may be responsible for small, structural alterations that block binding of both phalloidin and jasplakinolide to F-actin. It should be noted that the amino acid contact points for jasplakinolide are currently undefined, so inferring a common mechanism of phalloidin and jasplakinolide binding is speculative. Alternatively, the loss of phalloidin binding and jasplakinolide sensitivity could be the result of the accumulation of multiple differences in residues that result in a global F-actin conformation change. These F-actin ultra-structural changes may then result in a faster dissociation rate for phalloidin and jasplakinolide in *A*. *fumigatus*, as previously noted [42, 47]. As a third possible scenario, the phalloidin / jasplakinolide binding pocket could be occluded in *A*. *fumigatus* by an *Aspergillus*-specific actin-binding protein. Since a complete identification of actin-binding proteins in *A*. *fumigatus* is lacking [2], this possibility cannot be ruled out. However, because the SCact1 strain grows normally and is phalloidin / jasplakinolide positive, such a protein would be of little functional importance to the *A*. *fumigatus* actin cytoskeleton.

In conclusion, our data show that minor differences in the actin sequences between yeast and *Aspergillus* actin proteins account for loss of phalloidin binding and jasplakinolide sensitivity. In addition, altering F-actin dynamics by pharmacological stabilization with jasplakinolide, or introduction of human β-actin, is detrimental to *A*. *fumigatus* viability. The *Scact1* strain, generated here, will be a useful tool for further dissection of how actin dynamics support fungal morphogenesis, including the interplay of signal transduction and stress responses with actin dynamics. Further delineation of the biophysical properties underlying differential sensitivity of human and filamentous fungal actins to pharmacological stabilization may lead to novel compounds for selective inhibition of fungal invasion.

## Supporting Information

S1 Fig Effects of temperature and media composition on *Scact1* and *HsactB* growth. Conidia from the wild type and *Scact1* strains were point inoculated onto GMM or YPD media, as described in the Materials and Methods (top panel). Agar plugs from the peripheral growth zone of wild type and *HsactB* cultures were transferred to fresh GMM or YPD media (bottom panel). All culture plates were subsequently incubated at either 30°C or 37°C for 3 days and colony diameter was scored every 24 hours. Growth rate was calculated as by the following formula: (Colony diameter in mm Day 1 –Colony diameter in mm Day 2) / 24 hours. Data represents the average of 3 samples ± S.D.
(PDF)
