# Supplementary material for: Differential Support of Aspergillus fumigatus Morphogenesis by Yeast and Human Actins
Source: PLoS One. 2015 Nov 10;10(11):e0142535. doi: 10.1371/journal.pone.0142535 (PMC4640809; doi:10.1371/journal.pone.0142535)
Supplement: S1 Fig — Conidia from the wild type and Scact1 strains were point inoculated onto GMM or YPD media, as described in the Materials and Methods (top panel). Agar plugs from the peripheral growth zone of wild type and HsactB cultures were transferred to fresh GMM or YPD media (bottom panel). All culture plates were subsequently incubated at either 30°C or 37°C for 3 days and colony diameter was scored every 24 hours. Growth rate was calculated as by the following formula: (Colony diameter in mm Day 1 –Colony diameter in mm Day 2) / 24 hours. Data represents the average of 3 samples ± S.D. (PDF) [file pone.0142535.s001.pdf]

S1 Figure. Effects of temperature and media composition on *Scact1* and *HsactB* growth.

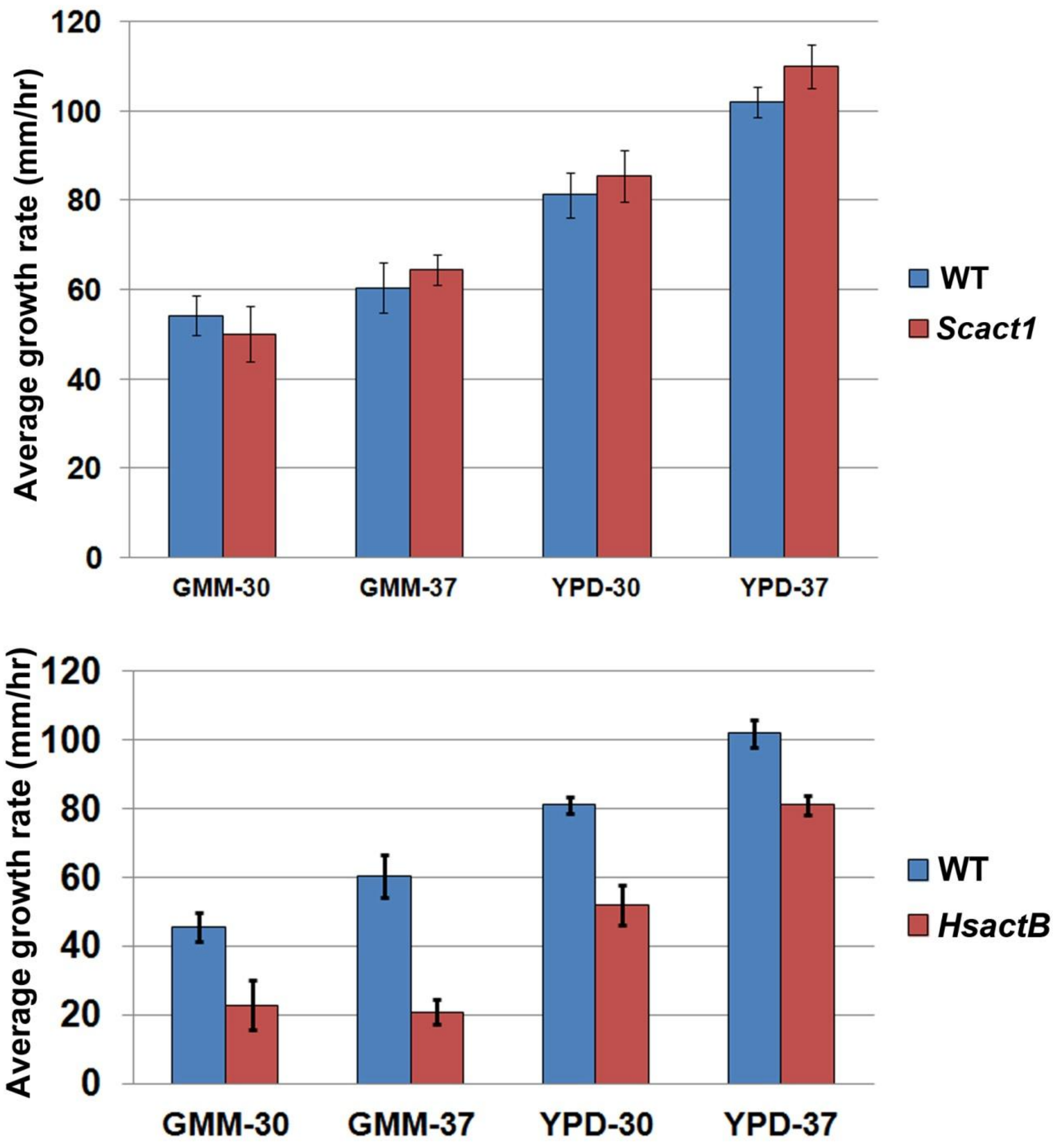

S1 Fig. Conidia from the wild type and *Scact1* strains were point inoculated onto GMM or YPD media, as described in the Materials and Methods (top panel). Agar plugs from the peripheral growth zone of wild type and *HsactB* cultures were transferred to fresh GMM or YPD media (bottom panel). All culture plates were subsequently incubated at either 30°C or 37°C for 3 days and colony diameter was scored every 24 hours. Growth rate was calculated as by the following formula: (Colony diameter in mm Day 1 – Colony diameter in mm Day 2) / 24 hours. Data represents the average of 3 samples ± S.D.
